# Megaproject Environmentally Responsible Behavior in China: A Test of the Theory of Planned Behavior

**DOI:** 10.3390/ijerph19116581

**Published:** 2022-05-28

**Authors:** Linlin Xie, Mian Huang, Bo Xia, Martin Skitmore

**Affiliations:** 1 School of Civil Engineering & Transportation, South China University of Technology, Guangzhou 510641, China; llxie@scut.edu.cn; 2 School of Architecture and Built Environment, Queensland University of Technology, Brisbane, QLD 4001, Australia; paul.xia@qut.edu.au; 3 Faculty of Society and Design, Bond University, Gold Coast, QLD 4229, Australia; mskitmor@bond.edu.au

**Keywords:** megaprojects, environmentally responsible behavior, theory of planned behavior, PLS-SEM, China

## Abstract

Construction megaprojects play a significant role in today’s infrastructure provision in terms of sustainable development, and their increasing proliferation worldwide means the environmentally responsible behavior (ERB) of those involved are becoming of vital importance. This study investigates how ERB can be best supported in megaprojects by first identifying the motivational factors that are involved based on the theory of planned behavior (TPB), followed by a survey of 188 managers involved in China megaprojects to test the TPB model using Partial Least Squares Structural Equation Modeling (PLS-SEM). The results strongly support the TPB model’s predictive ability, with subjective norms being the strongest predictors, followed by attitudes and perceived behavioral control. These findings provide support for decision makers in helping to cultivate and improve the level of megaproject ERB in China and many other such countries that are similarly involved.

## 1. Introduction

Megaprojects, with their huge spatial and temporal characteristics have been significant drivers and boosters of economic and social development [1]. Thus, both public and private organizations worldwide have resorted to the development of megaprojects as one of the most preferential routes to provide benefits to local municipalities and society at large [2]. However, they are typically characterized by “enormous resource consumptions and significant environmental impacts” [3,4,5]. In most cases, megaprojects, especially in the construction industry, can cause serious environmental problems [3,6], as evidenced by the environmental and ecological damage caused by The Baram River Dam and the Karakum Canal [7,8], and the social and environmental impacts of the Qinghai-Tibet Railway on the Tibetan Plateau [8]. Environmental protection has therefore become one of the most important concerns of megaproject organizations [9]. 

The study of megaproject environmental responsibility is of great theoretical and practical significance as it is useful for promoting the high-quality development of projects and improving the sustainability of ecological resources [10]. This is specifically related to the problem of environment, natural resources, and ecological protection faced by management in the construction and operation of engineering projects, such as in pollution prevention and control, soil and water conservation, ecological balance, and the protection of endangered flora and fauna [11]. Previous research into megaproject environmental responsibility mainly focuses on environmental impact assessments [12,13], environmental management procedures [14], and environmental management strategies [15], which reflects the amount of attention and concern it has attracted.

Although the importance of promoting environmentally responsible practices is widely recognized in megaprojects, the key challenge in practice is to translate formal project management strategies into practical initiatives. In addition, very limited research has sought to integrate an organization’s macro-level environmental strategies into environmentally responsible behavior (ERB) at the behavioral and psychological levels [5]. ERB is defined as a series of behaviors that reduce adverse impacts on the environment and promote environmental protection [16]. It reflects the effectiveness of existing environmental protection strategies, which determines the final quality of environmental performance. It is of importance, therefore, to urge construction megaproject participants to promote and implement effective ERB in the global context of sustainable development [6].

The literature presents some of the major variables that impact on environmental responsibility practice. Previous studies mainly focus on the factors influencing the formation of ERB by permanent organizations, such as environmental awareness [17], perceived benefits [18], regulatory pressure, and social pressure [19]. In recent years, there has been an increasing number of similar studies of temporary project organizations, identifying such contributing factors as knowledge, innovation, and regulation [20,21,22]. It is clear that megaproject management involves a wider and more complex range of stakeholders than corporate operations or general construction projects [23]. The implementation of megaprojects’ ERB relies on the close collaboration between diverse and heterogeneous participants throughout the project lifecycle [24]. The decisions they make and the actions they take have different, sometimes mixed, motivations. 

However, a deep understanding of the motivational factors that affect megaproject ERB has been hitherto lacking. Wang et al. focus on elaborating the influence of project organizations’ environmental responsibility on organizational citizenship environmental behaviors by practitioners [5], with a later emphasis on the environmental protection practices of peer projects as well as expert norms [25]. However, research is limited to individual informal environmental behaviors (i.e., organizational citizenship environmental behaviors) and does not cover all dimensions of the megaproject ERB. Therefore, the conclusions may not be applicable to megaproject ERB. Xue et al. verify the positive impact of formal and informal relationships on megaproject environmental protection practices [9]: their survey considers only interorganizational relationships, but fails to fully explore the influence of social-psychological and social-environmental factors [26].

This paper aims to investigate the determinants of megaproject ERB in China. Since ERB is rational behavior and is likely to be influenced by various factors such as psychological, institutional, and capacity, the theory of planned behavior (TPB) is adopted for investigation. It is expected that the factors, together with their revealed interrelationships, will deepen the understanding of megaproject ERB. Meanwhile, the findings will enable decision makers to design policies for enhanced megaproject environmental responsibility practices.

The remainder of this article comprises a literature review of related studies in Section 2, followed by the theoretical basis and research hypotheses in Section 3. Section 4 describes the research methodology and Section 5 provides the data analysis results, while Section 6 discusses the research results prior to the final concluding section.

## 2. Literature Review

### 2.1. Megaprojects and ERB

Compared with ordinary engineering projects, a megaproject is a huge spatial entity. With the development of the construction plant manufacturing model, the supply chain of bulk resources at the project site presents a large spatial distribution structure. Therefore, the wide geographic space that is involved leads to a huge scope in terms of influence and radius of action. The scope of environmental responsibility may be gradually increased beyond the geographic space of the project itself and expand to a wider spatial area, causing more serious environmental problems [10].

The number of megaprojects has continued to increase in recent years and the environmental issues arising from their construction activities have attracted global attention [25]. They are required to advance environmental management in an effective and responsive manner [27]. Several aspects of megaproject environmental responsibility management have been studied, including exploratory research into such topics as supply chain management [28,29]; green building [20]; construction waste management [30,31]; environmental impact assessment [13,32]; environmental management systems [14]; and environmental regulations [33] However, the literature often focuses on a single aspect of environmental protection. The fragmented research cannot adapt to the reality of the needs of megaproject management.

Numerous studies have shown that enhancing ERB is an effective method to reducing harm to natural environments for both companies and individuals [34,35]. With the increasing emphasis on sustainability, the study of ERB (a key element in promoting environmental protection practices in megaprojects) has become even more necessary.

Studies have addressed topics related to ERB in detail, including theoretical ERB models [36,37,38]; factors influencing ERB [39,40,41,42]; the structure of corporate ERB [43,44]; the impact of corporate ERB on financial performance [45,46,47]; measurement of corporate ERB [16,48,49,50]; and the value created by corporate ERB [51,52]. Moreover, many publications address topics related to ERB in such specific disciplines as sustainable consumption [53]; environmental protection in tourism [54]; energy efficiency [55]; marketing [56]; green innovation [57,58]; and information systems [59]. 

Although individual pro-environmental behavior and corporate ERB have been considered in several studies of green practice issues, much about megaproject ERB still remains to be examined empirically. Unlike ERB being undertaken by a single company or individual, the implementation of megaproject ERB relies on the close collaboration of diverse and heterogeneous participants throughout the project’s life cycle. They have diverse and sometimes mixed motives for the decisions they make and the actions they take. Thus, it is important to identify the key factors that motivate and support megaproject ERB in these organizations and understand how these factors operate.

### 2.2. Drivers of Megaproject ERB

In the context of megaprojects, ERB means integrating the concept of environmental protection into project investment and construction activities, controlling pollution, and saving such resources as strategy, organizational structure, culture, and production from all aspects of the project. Clearly, it is unrealistic to solely rely on the project organization’s integrity to fulfill its environmental responsibility—it is necessary to further study the mechanism driving the active adoption of environmental responsibility from the perspective of the project.

Academic research into construction projects has investigated the factors influencing environmental behaviors. In terms of internal factors, Yusof et al. argue that construction companies’ energy efficiency and waste management practices have a positive influence on their employees’ environmental behavior [60]. Li et al., for example, identify the project team’s knowledge and skills as key drivers [33], while such factors as innovation and technological development also occur in other studies [20,22]. As for external factors, Fu et al. identify government agencies’ monitoring and incentives during the lifecycle of construction projects as directly influencing the initiative of stakeholders’ green behavior [61], with Wong et al. finding mandatory government environmental regulations, client requirements in tenders, and NGO requirements to be critical factors for the green procurement of construction projects [21]. In addition, studies have integrated both internal and external drivers, with Jain et al., for example, finding contractors’ construction waste-recycling behavior to be influenced by a combination of perceived benefits, perceived costs, environmental awareness, and regulatory pressure [62].

Although the literature considers the influence of internal and external factors in terms of firm or specific environmental behaviors, little is known of the essential reasons for the active adoption of megaproject environmental responsibility. Xue et al. verify the positive impact of formal and informal relationships on megaproject environmental protection practices from the perspective of interorganizational relationships [9].

Compared with enterprises or general construction projects, the environmental management of megaprojects involves a wider and more complex range of stakeholders [11], between which the complicated relationships require an appropriate balance of interests [27]. In addition, the environment surrounding a megaproject is a complex self-organizing system: during the project’s whole lifecycle, environmental behavior is dynamic and exhibits complex self-organization and self-adaptation characteristics, which is a form of uncertainty phenomenon with a complex and emergent mechanism. The characteristics of megaprojects dictate that ERB is more complex and sensitive than that of general projects. Megaprojects have a wider range of influencing factors and therefore an integrated perspective provides a better means to understand the factors that motivate and support the formation of their ERB.

### 2.3. Theory of Planned Behavior

The theory of planned behavior (TPB) is proposed by Ajzen based on the theory of rational behavior, which integrates various factors, including the actors, internal management of the organization, and external environment of the organization, and provides a systematic analysis framework for the analysis of individual or organizational behavioral intentions. It has received increasing attention and application in such fields as tourism, advertising, environmental management, and project management [63,64,65].

TPB has been well applied and developed in several construction project studies. Yuan et al., for example, use TPB to investigate the predictors of project managers’ intentions regarding waste reduction [66], and Yang et al. analyze developer green purchasing behavior based on TPB [67]. Zheng et al. adopt TPB to investigate the formation of interorganizational relationship behavior in megaproject construction [65]. Liu et al. explore the predictors of participants’ knowledge-sharing behavior based on TPB, and reveal how behavioral intention attitude, subjective norms, and perceived behavioral control affect the intention of the knowledge-sharing behavior in the megaproject construction [68]. Therefore, the interpretation of the TPB-based megaproject ERB is feasible.

The TPB states that attitudes, subjective norms, and perceived behavioral control combine to shape actors’ behavioral intentions and behaviors. In particular, attitudes in the TPB model refer to actors’ positive or negative evaluation of the effects of a specific behavior. Subjective norms reflect an actors’ perception of other participants regarding whether they should perform a specific behavior, and perceived behavioral control is related to the actors’ perception of the existence or absence of resources or opportunities necessary for the behavior [69].

**Figure 1:**
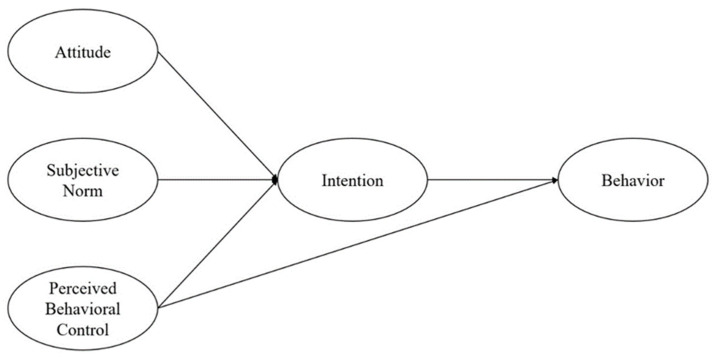
Theory of planned behavior.

In response, the present study introduces TPB into a research framework of megaproject ERB, involving Ajzen’s three influencing factors of behavioral attitude, subject norms, and perceived behavioral control (Figure 1).

## 3. Hypothetical Development

### 3.1. Attitude

Attitude is the degree to which a behavior is evaluated as good or bad. In this study, attitudes are defined as positive or negative attitudes that are held by megaproject organizations concerning whether to implement appropriate green management behaviors and assume appropriate environmental responsibilities. Decision makers who believe that favorable results will be obtained by adopting a particular behavior have a positive attitude toward that behavior [69]. Consistent with Osmani et al. [70], project teams have a positive attitude to ERB if they predict benefits or positive outcomes as a result. For megaprojects, ERB plays an important role in reducing energy consumption and enhancing environmental protection [5]. The active adoption of environmental behaviors can improve the reputation and market competitiveness of construction companies [67]. Participants usually need to hold a good image and satisfaction expectation [71]. The organization’s resulting improved environmental benefits and good reputation from ERB can continue to motivate its behavioral intentions [67]. Hence, it is posited

**Hypothesis** **1** **(H1).** *The project organizations’ attitudes positively influence the intention towards megaproject ERB.*

### 3.2. Subject Norms

According to the basic view of TPB theory, subjective norms refer to the awareness of megaproject participants of the social pressure to perform (or not perform) ERB. As demonstrated by Fishbein and Ajzen, when people are aware of and accept a given sociocultural norm, their behavior is likely to change. First, the environmental aspects of megaprojects have been scrutinized by environmental regulators due to their significant ecological impact. Project participants often experience changes in practice in response to stringent environmental audits and regulations [25]. It has been argued that participants are more likely to adopt environmental citizenship behaviors if they encounter the influence of government agencies [72]. Second, professional groups in the field of environmental protection often shape shared values, norms, and standards of expected behavior. These norms and collective expectations are diffused and developed through such information exchange activities as industry conferences, professional consultation, and vocational education. Industry experts, consulting firms, and research institutions require managers to pay attention to environmental protection issues and promote environmental protection behaviors [25]. Furthermore, ERB is influenced by frequent media reports on the project’s progress. Therefore, the subjective norms for adopting megaproject ERB can be measured by regulatory environment and socio-cultural environment variables.

**Hypothesis** **2** **(H2).** *Subjective norms positively influence the intention towards megaproject ERB.*

### 3.3. Perceived Behavioral Control

Perceived Behavioral Control (PBC) refers to the ability of an organization (including such elements as knowledge, competence, and control) to actually implement specific behaviors [69]. PBC in this study concerns megaproject participants’ perceptions of the difficulty in adopting ERB. The more an organization believes it has the necessary resources or capabilities to perform a given behavior, the more likely it is to intend to and actually perform that behavior at a later date [69]. Moreover, the practitioners’ perceptions of the difficulty of adopting ERB are affected by economic feasibility [73], technical feasibility [66], and policy support [74]. Construction projects need to consider cost targets and megaproject environmental practices may require high levels of funding [10]. In China, cost control is the biggest challenge to implementing green practices [75], while research in Singapore shows the experience and skill level of construction practitioners to be a key factor affecting the productivity of green buildings [20]. Advanced building information technology can promote green design and reduce material waste [21]. Policy support is another driver of environmental practices [21,76]. As Tang and Ng have found, government incentives influence the vision of construction firms, thereby changing their sustainability strategies, with explicit government subsidy policies also having a significant impact on the processes and outcomes of both emerging and established firms [77]. Hence, we propose

**Hypothesis** **3** **(H3):** *PBC positively influences the intention towards megaproject ERB.*

According to TPB, PBC can influence behavioral intentions and directly predict actual behavior. When an individual’s perception of behavioral control accurately reflects his or her actual control conditions it can directly predict the likelihood of the behavior. When participants can accurately control their actual future behavior through perceived control ability, perceptual control ability directly predicts the possibility of the participant’s ERB, i.e., PBC directly affects participant ERB. Therefore, we propose

**Hypothesis** **4** **(H4).** *PBC positively influences ERB in megaprojects.*

### 3.4. Behavioral Intention

According to TPB, behavioral intention reflects the motivation for behavior and is its immediate antecedent [69]. It mediates the relationship between attitude and behavior between subjective norms and behavior, and between perceived behavioral control and behavior [69,78]. The influence of behavioral intentions on actual behavior has been recognized in many different domains, including relationship behavior [65] and technology adoption [79]. Armitage and Conner validated the intentional behavior pathway based on a meta-analysis of 185 published studies [80]. Focusing specifically on studies of environmental behavior, environmental behavioral intentions have been found to serve as predictors and proxy variables for the likelihood of environmental behavior. In the context of construction projects, the behavioral intentions of project participants are also considered to have a direct influence on ERB [81,82]. We therefore propose

**Hypothesis** **5** **(H5).** *Behavioral intention positively influences ERB in megaprojects.*

## 4. Research Methodology

### 4.1. Research Instrument

The process of measurement development began with an investigation of the theoretical and empirical literature of ERB and megaprojects. The measurement items that were used for the constructs were primarily developed based on existing scales from the extant literature that have been proven reliable and were modified to fit the megaproject context. All constructs were measured reflectively with multiple items on five-point Likert scales.

A questionnaire was vetted by two experts and subjected to a pilot study with 23 experienced practitioners (with over 5 years of experience). An examination of the results of the pilot study led to further refinement, including removing four redundant items, combining two that overlapped, and rephrasing items that were considered confusing. The final version of measurement items is provided in Table 1.

Pre-procedural remedies were adopted to minimize response bias. First, the anonymous online questionnaire was selected as the primary approach to collect the data since this self-administered method proved to be valid for reducing the likelihood of bias due to there being less social interactions and assured anonymity [9]. Second, the respondents were asked to complete the survey based on their most recently experienced megaproject and informed that there was no wrong answer. As a result, they could provide a relatively clear description of the environmental responsibility practices involved and thereby avoid preferentially selecting their most successful experiences, which ultimately reduced the risk of providing socially desirable responses. Third, the survey items under general topics were distributed rather than grouped by construct, thereby reducing the inertia of respondents in answering questions. Finally, a question (Are you familiar with the project’s environmental activities?) was included with the response options of Yes, No, or Unsure. Only respondents who provided a definite answer of Yes were retained, while the No or Unsure answers were filtered out [83].

### 4.2. Unit of Analysis and Survey Procedure

The data were collected from Chinese megaprojects. Because China is experiencing its “biggest infrastructure investment boom” in recent years, a large number of such projects have provided first-hand data for previous empirical surveys [85]. In this study, we aimed to distribute the questionnaire to diverse respondents from various megaprojects of different project types to increase the representativeness of the overall sample and provide a broader view of industry practice.

According to Wang et al., a megaproject is defined as a large-scale infrastructure costing over CNY 1B, and which significantly affects social production, economic growth, individual livelihoods, and the natural environment [83].

The questionnaire survey was formally conducted between May 2021 and August 2021 in China. Only professionals who were directly involved in megaproject environmental practices were considered as eligible targeted respondents. These professionals should be familiar with environmental laws, regulations, and policies, and have previous experience in environmental activities.

After removing the short response-time responses or invariant responses in a row, 188 valid eligible responses remained for the analysis. The surveyed megaprojects comprise 79.79% of public projects, 19.15% of public-private partnership projects, and 1.06% of private projects. Demographic characteristics of the projects and respondents are summarized in Table 2.

### 4.3. Statistical Analysis

Partial Least Squares Structural Equation Modeling (PLS-SEM) is one of the most frequently used techniques to analyze causal models, and has also been used in the field of megaprojects [83]. PLS-SEM is appropriate when the research goal focuses on maximization of the explained variance in endogenous constructs, identification of important “driver” constructs, or extension of a theory [86]. PLS analysis techniques do not involve any data distribution assumptions and can work effectively with small sample sizes [86,87]. PLS-SEM is therefore used to test the conceptual model, as the primary objective of the present study is to explain the variance of the key target construct (MERB) caused by the other constructs (AT, SN, and PBC). The sample size is small, and the data are not normally distributed.

**Figure 2:**
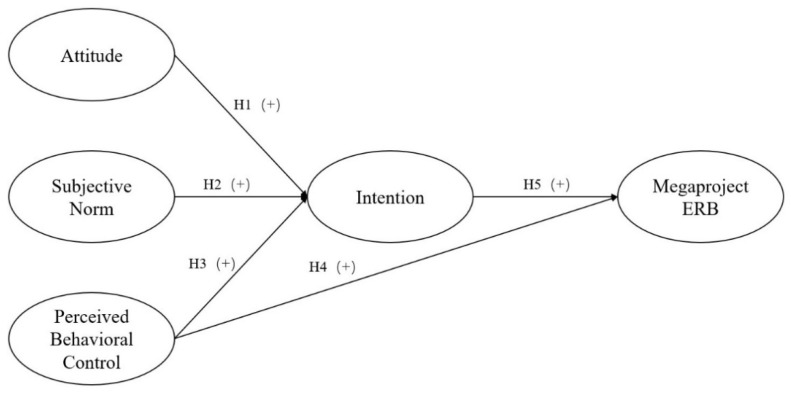
Conceptual model.

For the data analysis, the SmartPLS v.3.0 software (SmartPLS GmbH, Bönningstedt, Germany) is used to evaluate the measurement scales and to test the research hypotheses. PLS-SEM allows the validity of a latent variable with its associated indicators (measurement model) and the structural relationship among latent variables (structural model) to be simultaneously examined [86,87]. The model comprises two parts, namely measurement and the structural model. Figure 2 describes the structural model, including the constructs and their connecting paths.

## 5. Results

### 5.1. Measurement Models

Before hypothesis testing, the variables need to be validated as correctly defined and measured. According to the previous study on PLS-SEM [88], the measurement models are examined via the indicator loadings, internal consistency reliability, convergent validity, and discriminant validity. Table 3 shows the test results, with the factor loadings of all the constructs ranging from 0.720 to 0.874, which meets Hair et al. criterion [88]. The internal consistency reliability is assessed using composite reliability (CR) and Cronbach’s alphas, and Table 3 shows these to be above the 0.7 threshold for all the constructs, which indicates that the corresponding scale has satisfactory internal consistency. Table 3 also shows convergent validity, with the average variance extracted (AVE) being more than the minimum requirement of 0.5. The discriminant validity is measured in two ways. First, as Table 4 shows, the value of the square root of AVE for each construct in the diagonal of the Fornell-Larcker criterion matrix is higher than any other values of its correlated constructs. Second, as Table 5 shows, each item’s loading on its construct is higher than any of its cross-loadings with other constructs. 

### 5.2. Structural Model

The structural model is evaluated by examining the coefficient of determination (R^2^), the blindfolding-based cross-validated redundancy measure Q^2^, and the statistical significance and relevance of the path coefficients. 

R^2^ is referred to as ‘in-sample predictive power’ and measures the proportion of the variance in the endogenous constructs. Acceptable R^2^ values depend on the context [88]. Construction management studies follow the suggestions of Chin in that R^2^ values of 0.19, 0.33, or 0.67 are considered “weak”, “moderate”, and “substantial”, respectively [89]. As Table 6 shows, the R^2^ value of IN is 0.644 and the R^2^ value of MERB is 0.633, which substantiates the model’s predictive validity. 

The value of Q^2^ is used to assess the PLS path model’s predictive accuracy. As the results in Table 6 show, each dependent variable is over zero which means that the structural models have predictive relevance.

**Figure 3:**
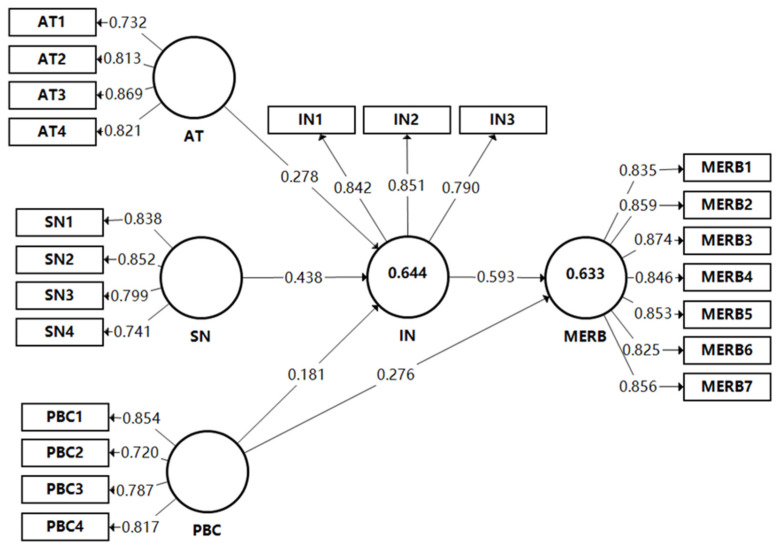
Results of the PLS analysis for the structural model.

The bootstrapping resampling procedure with 188 cases and 5,000 subsamples is conducted to determine the path significance, which is used to test the hypothesis formulated based on the model. Figure 3 and Table 6 show the results, indicating the path coefficients between AT, SN, PBC, and IN to be β = 0.278 (*p* < 0.05); β= 0.438 (*p* < 0.001); and β = 0.181 (*p* < 0.05), supporting hypotheses H1, H2, and H3. For H4, the path coefficient (β = 0.276, *p* < 0.001) is significant at the 0.1% level: therefore, this finding provides confirmation that the PBC will positively influence MERB. Meanwhile, the IN and MERB link is significant (β = 0.593, *p* < 0.001. Hence, both Hypotheses 4 and 5 are supported.

## 6. Discussion

This study investigates ERB in the context of megaprojects based on the theory of planned behavior. The results support the hypotheses linking attitudes, subjective norms, and perceived behavioral control to intentions and ERB, suggesting that attitudes, subjective norms, and perceived behavioral control (competence) play a determining role in fostering ERB.

### 6.1. Major Findings

The research findings suggest that behavioral attitudes have a positive impact on behavioral intentions towards megaproject environmental responsibility (H1 in Table 5). This is consistent with such studies of environmental protection in the construction industry as Begum et al. [90] and Li et al. [82], and indicates that participants who perceive benefits from ERB are more willing to adopt ERB. Participants focused on accomplishing product goals during project construction also need to consider creating sustainability in a complex economic and social environment. The project participants will consider whether it is profitable to actively manage environmental issues [91]. Environmental protection is critical to the success of any business or project [92]. From this perspective, therefore, participants should consider enhancing project performance and benefits through an improved intent towards ERB.

Subjective norms positively influence megaproject ERB, supporting hypothesis H2. This finding corresponds with other studies that find that regulatory and social pressures strengthen the environmental behavioral intentions of construction project participants [73,93]. It is worth noting that subjective norms overtake behavioral attitudes and perceived behavioral control as the dominant factor in shaping behavioral intentions. This is slightly different from Yuan et al. who explicitly state that attitudes are the most critical predictor of Chinese project managers’ waste reduction intentions, although they also recognize the importance of subjective norms, and perceived behavioral control in influencing waste reduction intentions [31]. A possible explanation for this result is that projects face significant environmental pressure from different external stakeholders (e.g., government regulators, industry associations, and the media) due to the current increasing attention paid to environmental sustainability. The pressure from external parties should also prompt project participants to pay more attention to ERB.

Consistent with other TPB-based studies, perceived behavioral control is considered to be an important motivator for promoting behavioral intentions and organizational behavior [67] (H3 and H4 in Table 5). These findings imply that sufficient resources and capabilities help to form participants’ intentions towards, and improve their level of, ERB. Participants are mostly employed by large, highly qualified units, and can provide support for ERB [67,90]. Furthermore, megaprojects in China are usually initiated by the central or local government and have a close relationship with them. As the main implementor of the national sustainable development strategy, the government can provide motivational support for ERB in construction work, particularly in the form of incentives [94].

Behavioral intention has a significant effect, i.e., H5 is verified. This result indicates that the higher the intention of adopting ERB by megaproject participants, the more they choose such behaviors. This is consistent with the results of such previous studies as Li et al. [82], for example, who argue that behavioral intentions can help to predict the construction waste-reduction behaviors of employees in construction firms.

### 6.2. Theoretical Contributions

This study makes two main theoretical contributions. First, the research findings extend prior studies of megaproject environmental management which focus only on the motivations of informal individual pro-environmental behavior. However, in the context of construction projects, many environmental behaviors are not individual, but collective. This study considers both formal and informal environmental behaviors and then analyzes the drivers from a project organizational perspective to provide an increased understanding of the predictors of environmental management practices.

Second, although previous studies show that behavioral motivations differ in improving environmental protection, hitherto, there has been no comprehensive framework incorporating various psychosocial incentives to predict ERB. In response, the present study extends the application of TPB to explain the ERB of participants, with the empirical results indicating that attitudes, subjective norms, and perceived behavioral control all influence organizational intentions, which in turn promote ERB.

### 6.3. Implication for Practice

There are a growing number of megaprojects, especially in developing countries [83], and their construction has a huge impact on the environment [95]. It is therefore important to improve their level of ERB. The findings of this study have three effective implications for promoting the adoption of megaproject environmental management practices.

First, the organizations that are involved need to be aware of the benefits of ERB at the outset: because ERB is influenced by perceived attitudes toward the benefits involved, it is important that project participants clearly understand the value of ERB, such as in gaining legitimacy, environmental management performance, and reputation. Top management teams need to consider strategies that enhance positive attitudes toward ERB by all participants and reinforce and control perceived attitudes throughout the project lifecycle to ensure the adoption of environmental management strategies.

Second, it is necessary to strengthen external pressure. Government regulations, industry supervision, and public concern play a significant role in promoting the practice of environmental responsibility. Government departments can enhance environmental protection supervision and exert pressure on the project management team to realize the importance of environmental issues. Such industry members as industry experts, consulting firms, and academic groups also need to be involved in project decision-making to exert a greater influence on project management. Moreover, the government should continue to increase the public’s environmental awareness and promote public participation in environmental management activities to help the management team to practice better environmental protection, according to local development needs.

Finally, the ability of ERB is an important factor in promoting the adoption of megaproject environmental management strategies. Based on the aforementioned studies, it can be concluded that perceived behavioral control has a significant effect on both the intentional and actual adoption of ERB. Although the high cost hinders construction companies from implementing ERB, higher qualified agencies can provide better green support for ERB, and hence project owners should consider their ERB capabilities when selecting the contractors.

## 7. Conclusions

Megaprojects have a huge and far-reaching impact on the environment. In a global context that is increasingly focused on environmental sustainability, they need to assume the important responsibility of environmental protection [92]. ERB is a key factor in promoting the effective utilization of resources and protecting the ecological environment [96]. However, although megaprojects’ ERB is influenced by various factors, research into the mechanisms behind these factors is hitherto lacking. To address this research gap, this study explores the key determinants of megaproject ERB; TPB is used as the underlying theoretical structure, namely attitudes, subjective norms, and perceived behavioral control. Data collected from a questionnaire survey of 188 experienced megaproject managers are analyzed using PLS-SEM to test five hypotheses.

Overall, the results strongly support the TPB model’s ability to predict megaproject ERB. The adoption of megaproject ERB is driven by both perceived behavioral controls and behavioral intentions. In particular, the results show that subjective norms are the strongest predictors of behavioral intentions for megaproject environmental responsibility, followed by attitudes and perceived behavioral controls. These findings imply that enhancing government regulation, industry supervision, and public attention; promoting participant identification of the potential benefits of ERB; and strengthening their green capabilities will improve the level of megaproject environmental responsibility.

The study is limited by using cross-sectional data that was collected by a questionnaire survey. Future studies could use qualitative analysis (e.g., in-depth and longitudinal case studies) to further validate our results. The study is also restricted to megaprojects in China due to the differences in other cultural contexts. Further studies in other countries can be conducted to determine the applicability of our findings elsewhere. Moreover, although the TPB model successfully predicted megaproject ERB, the antecedent analysis was limited to three key dimensions. Therefore, it will be valuable to further analyze the factors influencing attitudes, subjective norms, and perceived behavioral control, and explore the potential interrelationships between these factors, which could provide a better understanding of their indirect or direct effects on megaproject ERB. Finally, the occurrence of extreme conditions such as the COVID-19 pandemic has affected construction practice in many different ways and warrants further research in the future to determine the applicability of the findings under extreme conditions.

## Figures and Tables

**Table 1:** Construct measurement.

Construct	Item	Key Source(s)
Attitude (AT)	AT1: Widely recognized by the state and society	[66]
	AT2: Easier to obtain construction awards	
	AT3: Get more market opportunities	
	AT4: Build a better image	
Subject norm (SN)	SN1: Requirements of government construction authorities	[66,76,84]
	SN2: Requirements of government environmental authorities	
	SN3: Expectations of local communities and the public	
	SN4: Initiatives of construction industry associations and environmental associations	
Perceived behavioral control (PBC)	PBC1: Technical assistance from the government	[21,33,66,75,76]
	PBC2: Project participants have a good understanding of environmental specifications and technologies	
	PBC3: Project participants have sufficient financial resources	
	PBC4: Project participants have extensive and skilled knowledge and management practices	
Intention (IN)	IN1: Intention at the beginning	[67,69]
	IN2: Intention to continue	
	IN3: Intention for the future	
Megaproject ERB (MERB)	MERB1: The environmental management system is perfect	[5,11,27,76]
	MERB2: Fulfillment of legal (regulations) and contractual obligations	
	MERB3: Emphasis on ecological and environmental protection	
	MERB4: Emphasis on environmental protection in residential communities	
	MERB5: Have a construction waste management plan	
	MERB6: Pay attention to the rational use of resources and reduce resource wastage	
	MERB7: Create conditions for the improvement of employee environmental awareness and skills	

**Table 2:** Demographics of the surveyed sample.

Variables	Category	Number	Percentage (%)
Project role	Government	4	2.13
	Owner	21	11.17
	Designer	2	1.06
	Contractor	136	72.34
	Supervisor	18	9.58
	Consultant	7	3.72
Position	Project manager	28	14.89
	Department manager	49	26.07
	Project engineer	111	59.04
Project investment (CNY 100 million)	10–20	91	48.41
	20–30	33	17.55
	30–40	11	5.85
	More than 40	53	28.19
Project duration	Less than 3 years	64	34.04
	3–4 years	89	47.34
	More than 4 years	35	18.62

**Table 3:** Convergent validity and internal consistency reliability.

Latent Variable	Items	Loadings	Cronbach’s Alpha	Composite Reliability	AVE
Attitude	AT1	0.732	0.824	0.884	0.656
	AT2	0.813			
	AT3	0.869			
	AT4	0.821			
Subject norm	SN1	0.838	0.823	0.883	0.654
	SN2	0.852			
	SN3	0.799			
	SN4	0.741			
Perceived behavioral control	PBC1	0.787	0.812	0.873	0.634
	PBC2	0.854			
	PBC3	0.720			
	PBC4	0.817			
Intention	BI1	0.842	0.770	0.867	0.686
	BI2	0.851			
	BI3	0.790			
Megaproject ERB	MERB1	0.835	0.936	0.948	0.722
	MERB2	0.859			
	MERB3	0.874			
	MERB4	0.846			
	MERB5	0.853			
	MERB6	0.825			
	MERB7	0.856			

**Table 4:** Discriminant validity (Fornell-Larcker’s criteria).

Latent Variable	AT	SN	PBC	BI	MERB
AT	0.810				
SN	0.740	0.809			
PBC	0.678	0.596	0.796		
IN	0.724	0.751	0.630	0.828	
MERB	0.700	0.743	0.649	0.766	0.850

Note(s): Figures in italic are the square root of AVE. AT: attitude; SN: Subject norm; PBC: Perceived behavioral control; IN: Intention; MERB: Megaproject ERB.

**Table 5:** Cross loadings for measurement items.

Code	Item Loadings				
	AT	SN	PBC	BI	MERB
AT1	0.732	0.658	0.385	0.547	0.586
AT2	0.813	0.548	0.493	0.554	0.513
AT3	0.869	0.585	0.651	0.634	0.568
AT4	0.821	0.611	0.645	0.606	0.600
SN1	0.556	0.838	0.404	0.639	0.653
SN2	0.614	0.852	0.471	0.658	0.632
SN3	0.556	0.799	0.488	0.610	0.545
SN4	0.690	0.741	0.594	0.508	0.571
PBC1	0.532	0.436	0.787	0.393	0.433
PBC2	0.588	0.547	0.854	0.616	0.611
PBC3	0.494	0.362	0.720	0.363	0.359
PBC4	0.542	0.512	0.817	0.562	0.593
IN1	0.604	0.572	0.597	0.842	0.628
IN2	0.613	0.602	0.539	0.851	0.625
IN3	0.580	0.688	0.429	0.790	0.648
MERB1	0.584	0.636	0.573	0.646	0.835
MERB2	0.602	0.638	0.576	0.704	0.859
MERB3	0.588	0.675	0.532	0.693	0.874
MERB4	0.584	0.616	0.501	0.635	0.846
MERB5	0.573	0.653	0.545	0.620	0.853
MERB6	0.638	0.587	0.545	0.625	0.825
MERB7	0.595	0.610	0.585	0.626	0.856

Note(s): AT: attitude; SN: Subject norm; PBC: Perceived behavioral control; IN: Intention; MERB: Megaproject ERB.

**Table 6:** Hypothesis testing results.

Variable	R^2^	Q^2^	Hypothesis Path	Path Coefficient	*p*-Value	Result
IN	0.644	0.426	AT > BI	0.278	0.001	H1: Supported
			SN > BI	0.438	0.000	H2: Supported
			PBC > BI	0.181	0.010	H3: Supported
MERB	0.633	0.450	PBC > MERB	0.276	0.000	H4: Supported
			BI > MERB	0.593	0.000	H5: Supported

Note(s): as Table 5.

## Data Availability

The raw data supporting the conclusions of this article will be made available by the authors, without undue reservation, to any qualified researcher.
